# Efficacy and safety of abacavir-containing combination antiretroviral therapy as first-line treatment of HIV infected children and adolescents: a systematic review and meta-analysis

**DOI:** 10.1186/s12879-015-1183-6

**Published:** 2015-10-26

**Authors:** Olatunji O. Adetokunboh, Anel Schoonees, Tolulope A. Balogun, Charles S. Wiysonge

**Affiliations:** Centre for Evidence-based Health Care, Faculty of Medicine and Health Sciences, Stellenbosch University, Cape Town, 7505 South Africa; Division of Community Health, Faculty of Medicine and Health Sciences, Stellenbosch University, Cape Town, 7505 South Africa

**Keywords:** Abacavir, Children, Adolescents, Antiretroviral therapy, HIV, Efficacy, Safety

## Abstract

**Background:**

Abacavir is one of the recommended nucleoside reverse transcriptase inhibitors (NRTIs) for the treatment of HIV infections among children and adolescents. However, there are concerns that the antiviral efficacy of abacavir might be low when compared to other NRTIs especially among children. There are also concerns that abacavir use may lead to serious adverse events such as hypersensitivity reactions and has potential predisposition to developing cardiovascular diseases

**Methods:**

We searched four electronic databases, four conference proceedings and two clinical trial registries in August 2014, without language restrictions. Experimental and observational studies with control groups that examined the efficacy and safety of abacavir-containing regimens in comparison with other NRTIs as first-line treatment for HIV-infected children and adolescents aged between one month and eighteen years were eligible. Two authors independently screened search results, extracted data and assessed the risk of bias of included studies using a pre-specified, standardised data extraction form and validated risk of bias tools. We also assessed the quality of evidence per outcome with the GRADE tool.

**Results:**

We included two randomised controlled trials (RCTs) and two analytical cohort studies with a total of 10,595 participants. Among the RCTs we detected no difference in virologic suppression after a mean duration of 48 weeks between abacavir- and stavudine-containing regimens (2 trials; *n* = 326: RR 1.28; 95 % CI 0.67–2.42) with significant heterogeneity (*P* = 0.02; I^2^ = 81 %). We also found no significant differences between the two groups for adverse events and death. After five years of follow-up, virologic suppression improved with abacavir (1 trial; *n* = 69: RR 1.96; 95 % CI 1.11–3.44). For cohort studies, we detected that the virologic suppression activity of abacavir was less effective than stavudine in both the lopinavir/ritonavir (1 study, *n* = 2165: RR 0.79, 95 % CI 0.67–0.92) and efavirenz sub-groups (1 study, *n* = 3204: RR 0.79, 95 % CI 0.67–0.92) respectively. The quality of evidence from RCTs was moderate for virologic suppression but low for death and adverse events, while that of cohort studies was low for all three these outcomes.

**Conclusions:**

Available evidence showed little or no difference between abacavir-containing regimen and other NRTIs regarding efficacy and safety when given to children and adolescents as a first-line antiretroviral therapy.

**Electronic supplementary material:**

The online version of this article (doi:10.1186/s12879-015-1183-6) contains supplementary material, which is available to authorized users.

## Background

The acquired immunodeficiency syndrome (AIDS) remains a major global concern with an estimated 3.3 million children and adolescents under 15 years of age currently living with the human immunodeficiency virus (HIV). Of these, about 2 million need antiretroviral therapy [1–3]. In order to effectively manage HIV infection and AIDS, it is recommended that antiretroviral treatment regimens should consist of a three-drug combination consisting of two nucleoside reverse transcriptase inhibitors (NRTIs) with either one protease inhibitor (PI) or a non-nucleoside reverse transcriptase inhibitor (NNRTI) depending on the age of the patient and other co-morbidities [4]. In the treatment of children infected by HIV, abacavir is one of the recommended NRTIs in children younger than 10 years of age [4]. Abacavir, formerly known as 1592U89, is a carbocyclic 2′-deoxyguanosine nucleoside analogue with its main activity being against HIV type 1 (HIV-1). It is phosphorylated to its active metabolite, carbovir triphosphate, which inhibits the HIV-1 reverse transcriptase competitively and terminates deoxyribonucleic acid (DNA) synthesis. This prevents HIV from replicating, thereby lowering the amount of HIV in the body’s system [5].

The two NRTIs in a three-drug antiretroviral regimen are referred to as the NRTI backbones of the regimen. The World Health Organization (WHO) recommends abacavir and either lamivudine or zidovudine as the NRTI backbones for children younger than 3 years of age [4]. There is a strong recommendation for the use of these two NRTI backbones as fixed-dose combinations in this paediatric age group; however, this recommendation was done with a low certainty of evidence [4]. In 3- to 10-year-old children and adolescents weighing less than 35 kg, abacavir-lamivudine is the NRTI backbone commonly used. Again, this regimen has strong recommendation for use but low certainty of evidence [4].

The WHO guidelines suggest that stavudine, a NRTI, be replaced by abacavir because of toxicity concerns [6, 7]. However, abacavir has adverse effect concerns of its own [8–11]. Abacavir is associated with a systemic illness known as abacavir hypersensitivity reaction that can result in death if the drug is not discontinued in affected patients. This hypersensitivity may present with fever, maculopapular rash and other constitutional symptoms such as fatigue, malaise and myalgia. Gastrointestinal adverse effects such as vomiting, diarrhoea and abdominal pain may also occur. Occasionally, there are also some prominent respiratory symptoms, such as tachypnea and cough [9, 11]. Hypersensitivity reactions due to abacavir have been reported in both paediatric and adult populations with the incidence in randomised controlled trials ranging from 0 to 14 % [11]. HIV-infected individuals of African descent seem to have reduced risk of abacavir hypersensitivity [12, 13], and the wide variation in reported adverse event incidence with abacavir use makes it necessary to do a systematic review, especially in children as there is a gap in the evidence-base. Furthermore, some cohort studies in South Africa have shown poor virologic responses to abacavir-based regimens when compared to stavudine in children. These studies queried the clinical effectiveness of abacavir when compared to the other NRTIs as well as the justification for making it a first-line drug in the treatment of HIV in children [14, 15]. A further investigation on the drug is thus needed.

Some research studies suggested that abacavir increases the risk of cardiovascular events, especially myocardial infarction [15, 16]. However, meta-analyses of randomised controlled trials in adults have not supported the postulation that abacavir-containing antiretroviral regimens carry a greater risk of cardiovascular events relative to abacavir-sparing regimens [17, 18]. Similarly, various studies evaluating changes in inflammatory and coagulopathic biomarkers upon commencement of abacavir-containing regimens have produced conflicting findings [19, 20]. These randomised controlled trials were carried out mainly on adults due to the belief that children have lower incidence of some of these important adverse effects of abacavir [21]. A meta-analysis of HIV infected adults switching to abacavir-containing regimens shows rather weak evidence of lower incidence of adverse events, with higher incidence of virological failure in the NRTI groups when compared to controls such as PI or NNRTI based regimens. [22].

Despite concerns that the confidence in the currently available evidence on the antiviral efficacy of abacavir might be low, coupled with possible serious adverse events such as hypersensitivity reactions and a potential predisposition to developing cardiovascular diseases, the WHO has recommended abacavir as one of the preferred NRTI backbones in the paediatric population [6]. We are not aware of any systematic review that assessed the efficacy and safety of abacavir-containing regimens in HIV infected children and adolescents.

The primary objective of this review was to assess the antiviral efficacy of abacavir-containing combination antiretroviral therapy (cART) regimens in comparison with cART regimens containing other NRTIs as first-line therapy for HIV infected children and adolescents.

The secondary objective was to assess the safety of abacavir-containing cART antiretroviral regimens in HIV infected children and adolescents.

## Methods

This review’s protocol has been registered in the PROSPERO International Prospective Register of systematic reviews (http://www.crd.york.ac.uk/PROSPERO/display_record.asp?ID=CRD42014009157), registration number CRD42014009157 and published in *Systemic Reviews* [23].

### Types of studies

Experimental [randomised controlled trials (RCTs) and non-randomised controlled trials] and observational studies with control groups were eligible for inclusion in this systematic review. Non-randomised controlled trials refer to studies that allocated participants to interventions and controls using alternation between groups, by the use of birth dates or weekdays or by other non-random methods.

### Types of participants

HIV infected individuals between 1 month and 18 years of age.

### Types of interventions

Experimental group: abacavir-containing cART regimens as first-line therapy.

Control group: cART regimens containing zidovudine or tenofovir or stavudine in the NRTI backbone as first-line therapy.

### Types of outcome measures

#### Primary outcomes

1. Virologic suppression. This was reported as the proportion of participants that reached a pre-defined concentration of HIV-1 RNA, typically <400 copies/mL or <500 copies/mL, at 48 weeks and/or 5 years. For purposes of meta-analysis we used the lowest reported value.
2. Virologic failure. This was reported as the proportion of participants who failed to suppress viral replication to non-detectable levels.

#### Secondary outcomes

1. Adverse events. We defined this as the proportion of study participants that required treatment interruption or switching.
2. CD4 cell count. We defined this as the mean change in the concentration of CD4 lymphocytes from baseline, as expressed in cells/μL.
3. Hypersensitivity reaction. We defined this as abacavir hypersensitivity reaction presenting with symptoms such as fever, nausea, respiratory discomfort, rash and diarrhoea.
4. Death (all cause).
5. Myocardial infarction and other cardiovascular events.

### Search methods for identification of studies

Regardless of language or publication status (published, unpublished, in press and in progress) we used a comprehensive search strategy to identify all relevant studies. This was done with the support of a health science librarian at the Faculty of Medicine and Health Sciences, Stellenbosch University, South Africa.

### Electronic databases

We searched the following electronic databases:

- MEDLINE via PubMed, on 8 August 2014
- Cochrane Central Register of Controlled Trials (CENTRAL), on 8 August 2014
- Scopus, on 9 August 2014
- ISI Web of Science (Science Citation Index), on 9 August 2014

Based on a search strategy published by Shey et al. [24], we used both text words and medical subject heading (MeSH) terms, for example abacavir, antiretroviral, HIV, *acquired immunodeficiency syndrome*, *child*, *paediatric*, *adolescent* and *randomized controlled trial* to form the basis of the search strings. We also used these terms in different combinations and with different spellings, and adapted them as appropriate for each database. Additional file 1 contains the detailed search strategies.

### Conference proceedings

We searched the proceedings of the following conferences for potentially eligible studies on 9 August 2014:

- The European AIDS Clinical Society (EACS) conferences
- International AIDS conferences
- Conference on Retroviruses and Opportunistic Infections (CROI)
- International AIDS Society conference on HIV Pathogenesis and Treatment (IAS)

We also searched for unpublished and ongoing studies in the following prospective clinical trial registries on 11 August 2014:

- ClinicalTrials.gov (https://clinicaltrials.gov/)
- WHO International Clinical Trials Registry Platform (http://apps.who.int/trialsearch/)

After having identified the included studies for this review, we contacted the relevant study authors to ask if they know of any other relevant studies in the field. We also screened the reference lists of included studies and relevant systematic reviews for additional studies.

### Selection of studies

Two review authors OOA and TAB, independently screened the titles and abstracts of all search outputs. We applied pre-specified eligibility criteria to identify potentially eligible studies. For these, we obtained the full text articles and again independently screened them for final eligibility. We provided reasons for excluding studies we viewed the full text of. Where needed, we contacted the authors of potentially eligible studies to obtain missing information or to clarify certain aspects of the study. We resolved disagreements by discussion and reaching consensus.

### Data extraction and management

OOA and TAB independently extracted study data using a standardised, pre-established data extraction form. We resolved disagreements by discussion and reaching consensus. For each included study, we extracted the following:

- Study details: contact details, citation, start and end dates, setting and design.
- Participant details: key eligibility criteria, ages, number of participants randomised per arm, losses to follow up, baseline HIV-1 RNA and CD4 cell levels.
- Interventions details: names of the drugs, doses and duration.
- Outcome details: virologic suppression, virologic failure, adverse events, CD4 cell count, hypersensitivity reaction to abacavir, death and cardiovascular events.
- Miscellaneous information: funding source and references to other relevant studies.

OOA entered the extracted data into Table 1 (Characteristics of included studies) and Table 2 (Characteristics of excluded studies).

**Table 1:** Characteristics of included studies

Studies	Brennan 2014 [29]	Musiime 2014 [28]	PENTA 2002 [31, 32]	Technau 2014 [14, 15]
Type of study	Prospective cohort study	Randomised controlled trial	Randomised controlled trial	Retrospective cohort study
Age range	5 to 14 years	1 month to 13 years	3 months to 16 years	4 to 129 months
Countries included	South Africa	Uganda Zambia	Belgium, Brazil, France, Germany, Ireland, Italy, Portugal, Spain and UK	South Africa
Period of enrollment	April 2009 to March 2011	May 2010 to September 2013	January 1998 to July 2000	August 1998 to April 2013
Sample size (n)	557	365	130	9543
Intervention	Abacavir, Lamivudine and Efavirenz	Abacavir, Lamivudine and NNRTI	Abacavir and Lamivudine ± Nelfinavir	Abacavir, Lamivudine and Lopinavir/ritonavir or Efavirenz
Comparators	Stavudine, Lamivudine and Efavirenz	Zidovudine, Lamivudine and NNRTI	Abacavir and Zidovudine ± Nelfinavir	Stavudine, Lamivudine and Lopinavir/ritonavir or Efavirenz
		Stavudine, Lamivudine and NNRTI	Lamivudine and Stavudine ± Nelfinavir	
Length of follow up	Up to 24 months	Up to 96 weeks	Up to 5 years	Up to 48 weeks
Funding sources	United States Agency for International Development (USAID)	Medical Research Council UK	European Commission, Medical Research Council, the Istituto Superiore di Sanità, Comunidad Autonoma de, Glaxo-Wellcome and Agouron	National Institutes of Health

**Table 2:** Characteristics of excluded studies

Study	Reasons for exclusion
Flynn 2014 [33]	Non-disaggregation of the results into different NRTI arms
Kline 1999 [34]	Having abacavir in all the arms
Musiime 2013 [35]	Measured outcomes are not of interest
Nahirya-Ntege 2011 [12]	Having abacavir in all the arms
Neely 2013 [36]	Having abacavir in all the arms
Penpact-1 2011 [37]	Non-disaggregation of the results into different NRTI arms
Sáez-Llorens 2001 [38]	Previously treated children

### Assessment of risk of bias in included studies

OOA and TAB independently applied the Cochrane Collaboration’s risk of bias tool ([25], Additional file 2) to assess the methodological quality of the included RCTs. This tool consists of seven domains, namely random sequence generation, allocation concealment, blinding of participants and personnel, blinding of outcome assessment, incomplete outcome data, selective outcome reporting and other forms of bias. We independently described our assessment for each domain by judging them alongside explanations as having either a ‘low risk’, ‘high risk’ of bias, or ‘unclear risk’ of bias.

To assess the methodological quality of the included cohort studies, OOA and TAB independently applied the Newcastle-Ottawa Scale (NOS) [26]. As per this tool, we judged each of the included cohort studies based on three broad categories, namely the selection of the study groups, the comparability of the groups, and the ascertainment of the outcome of interest. A study can be awarded a maximum of one star for each numbered item within the Selection and Outcome categories, and a maximum of two stars for the Comparability section. Detail appears in Additional file 3.

We compared our individual judgements and resolved discrepancies by discussion and reaching consensus.

### Measures of treatment effect

We used Review Manager 5.3 [27] to manage the data and to conduct analyses based on standard Cochrane Collaboration methods [25]. We analysed results for RCTs and observational studies separately. We calculated risk ratios (RR) for dichotomous data and planned to use mean differences (MD) for continuous data. However, none of the analysed data was of continuous nature. We presented all results with 95 % confidence intervals (CI).

### Unit of analysis issues

Individual participants was the unit of analysis in each of the included studies. The included RCTs had more than two intervention arms and we only compared those that are relevant and containing antiretroviral combinations that are currently in use. We did not include the same group of participants twice in the same meta-analysis. We analysed outcome data that were available for two or more time periods in separate meta-analyses.

### Dealing with missing data

We contacted the authors of Musiime 2014 [28] in September 2014 for clarity on non-disaggregated data between the treatment naïve and treatment experienced participants for outcomes such as adverse events, hypersensitivity reaction and death but did not get any response. Brennan 2014 [29] and Technau 2014 [15] were also contacted in September 2014 to obtain unreported data such as hypersensitivity reactions, CD4 cell counts and adverse events. However, Technau 2014 responded that we should use the available data while efforts would be made to send other needed information to us but we did not receive any of the requested additional information till the end of the review.

### Assessment of heterogeneity

We assessed heterogeneity between studies visually by inspection of forest plots and statistically by means of the Chi-square test where we pre-specified *P* < 0.1 to indicate significant heterogeneity. We also quantified heterogeneity with the I-squared (I^2^) test, where an I^2^ value of 50 % or more was taken to indicate substantial heterogeneity [25].

### Assessment of reporting biases

We planned to assess publication bias per outcome by means of funnel plots where at least 10 studies have been included. This review, however, did not have enough studies [25].

### Data analysis

We carried out all meta-analyses with the random-effects model because heterogeneity for the outcomes was substantial (I^2^ > 50 %); otherwise, we would have used the fixed-effect model. For outcomes with only one study where meta-analyses were not possible, a narrative synthesis was done.

### Subgroup analysis and investigation of heterogeneity

We planned to conduct subgroup analysis for different types of antiretroviral agents (PIs and NNRTIs), study settings (low, middle and high income countries) in the intervention and control groups, and to compare the intervention effects across different age groups (infants, children and adolescents) but the available data did not allow this.

### Sensitivity analysis

We planned to conduct sensitivity analyses to assess the effects of risk of bias and different statistical methods employed in the meta-analyses for the primary outcomes. However, due to the small number of included studies such analyses were not practical.

### Ethics

This study was a systematic review and meta-analysis of existing literature and did not involve any human subjects, therefore we did not require any approval from an ethics committee.

## Results

**Fig. 1:**
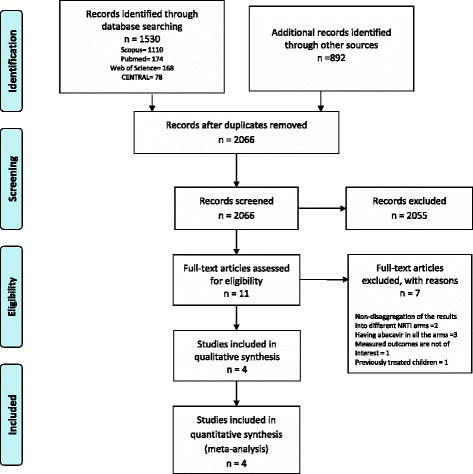
Flow diagram of the search and selection process for this review

Figure 1 displays the process for searching and selecting studies in accordance to the PRISMA guideline [30]. We screened 2066 records and identified 11 studies as potentially eligible.

The identified records were published in English, French, Spanish, French and German languages. We screened the non-English language abstracts by downloading their translated English version from the databases. After screening of the full-text articles of the potentially eligible studies, only four studies met the eligibility criteria. The rest were excluded with reasons as shown in Table 2. No ongoing studies were identified.

### Included studies

We included two RCTs [28, 31, 32] and two analytical cohort studies [14, 15, 29] with a total of 10,595 children. The studies were conducted in different countries across Europe, South America and sub-Saharan Africa. Table 1 gives additional details.

### Excluded studies

We excluded seven studies. Two studies were excluded due to non-disaggregation of the results into different NRTI arms [33, 34], three studies were excluded due to having abacavir in all the intervention arms [12, 35, 36] while another study was excluded because the participants were previously treated children [37]. A study was also excluded because the outcomes were not of interest [38]. Table 2 displays the reason for exclusion per study.

### Risk of bias and methodological quality assessment in included studies

We judged Musiime 2004 to have an unclear risk of bias because of incomplete statements concerning the participant selection process. PENTA 2002had low risk of selection bias as a result of adequate randomisation and allocation concealment. PENTA 2002 and Musiime 2004 were judged to have low risk of bias for blinding because the reported outcomes are objective and unlikely to be influenced by lack of blinding. The two studies were judged to have a low risk of attrition bias as they did not have differential or large numbers of losses to follow-up across the intervention arms. PENTA 2002 was judged free of selective reporting while Musiime 2004 was unclear because the available article is a conference presentation with limited information. The authors of PENTA 2002 reported having been sponsored by pharmaceutical companies and governmental agencies but gave reassurances that these organisations did not influenced the trial. The authors of Musiime 2002 did not report in the article the roles and involvement of the funders and we were unsuccessful to obtain this information from them via email. Brennan 2014 had three stars in the selection domain, one star in the comparability domain and two stars in the outcome domain while Technau 2014 had three stars in selection domain, one star in comparability domain and one star in the outcome domain. On a general note the cohort studies perform fairly well in the selection domain but poorly in the outcome domain.

Our judgements regarding the risk of bias in each included RCT and methodological quality assessment for cohort studies were presented in Tables 3 and 4.

**Table 3:** Risk of bias in included RCTs

	Random sequence generation	Allocation concealment	Blinding of participants and care providers	Blinding of outcome assessors	Incomplete outcome data	Selective reporting	Other bias
Musiime 2014 [28]	Unclear	Unclear	Low	Low	Low	Unclear	Unclear
PENTA 2002 [31]	Low	Low	Low	Low	Low	Low	Low

**Table 4:** Quality assessment of cohort studies

Quality evaluation	Brennan 2014 [29]	Technau 2014 yy
Representativeness of the exposed cohort	*	*
Selection of the non-exposed cohort	*	*
Ascertainment of exposure	-	-
Demonstration that outcome of interest was not present at the start of the study	*	*
Comparability	*	*
Assessment of outcomes	-	*
Was follow up long enough for outcomes to occur	*	-
Adequacy of follow up of cohorts	*	-
Total score	6	5

Table 4 shows the quality assessment of each of the included cohort studies using the Newcastle - Ottawa Scale. Each item can receive 1 star (*), except for Comparability that can receive 2 stars. The total number of stars represents the score, which demonstrates the quality of the study

### Effects of interventions

#### Comparison 1: Abacavir-containing cART regimens compared to cART regimens containing zidovudine or stavudine (RCTs)

##### Virologic suppression

**Fig. 2:**
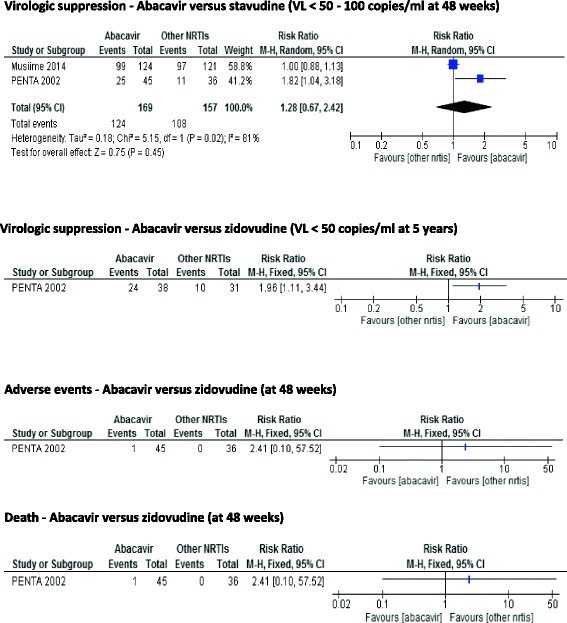
Forest plots of abacavir-containing regimen versus other nucleoside reversible transcriptase inhibitors (RCTs)

Musiime 2004 defined virologic suppression as <100 copies/mL concentration of HIV-1 RNA while PENTA 2002 defined virologic suppression as <50 copies/ml. We found no significant difference between abacavir and stavudine in virologic suppression: 2 RCTs, 326 participants with a RR of 1.28 (95 % CI 0.67–2.42). In Musiime 2004 where the participants belong to the low income countries / NNRTI subgroup, we detected no difference between the two groups (RR 1.00; 95 % CI 0.88–1.13; *n* = 245). In PENTA 2002 where the participants belong to high income countries/PI based subgroup, children on abacavir containing regimen were more likely to achieve virologic suppression than those on stavudine based regimen (RR 1.82; 95 % CI 1.04–3.18; *n* = 81; I^2^ for heterogeneity = 81 %) (Fig. 2).

**Fig. 3:**
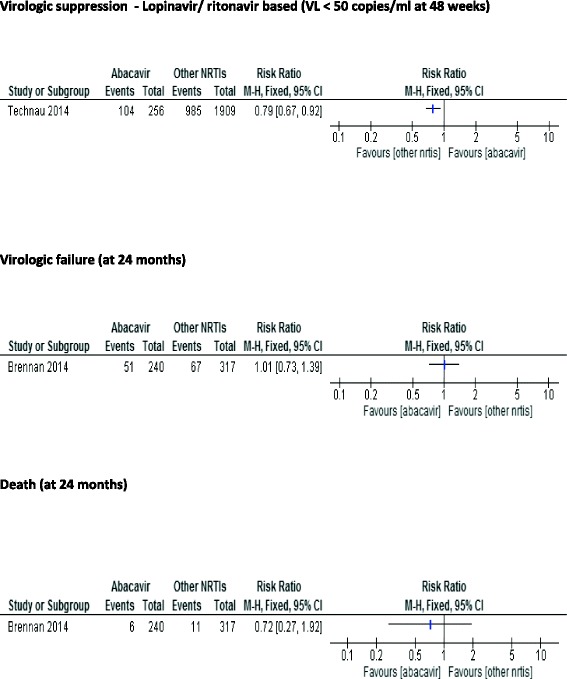
Forest plots of abacavir-containing regimen versus other nucleoside reverse transcriptase inhibitors (cohort studies)

We detected no difference between abacavir and zidovudine (2 RCTs, 325 participants) with a RR of 1.22 (95 % CI 0.55–2.72). Musiime 2004 reported less likely virologic suppression in the abacavir group (RR 0.89; 95 % CI 0.80–0.99; *n* = 244). In PENTA 2002, children on abacavir- containing regimen are likely to achieve undetectable virologic suppression than those on the zidovudine-containing regimen (RR 1.81; 95 % CI 1.04–3.18; *n* = 81; I^2^ for heterogeneity = 88 %. The quality of the evidence for this outcome was moderate as shown in Table 5.

**Table 5:** Summary of findings table for abacavir-containing cART regimens compared to cART regimens containing zidovudine or stavudine (RCTs)

Outcomes	Illustrative comparative risks* (95 % CI)		Relative effect (95 % CI)	No of Participants (studies)	Quality of the evidence (GRADE)
	Assumed risk	Corresponding risk			
	AZT or d4T - containing combination antiretroviral regimens	ABC- containing combination antiretroviral regimens			
Virologic suppression - ABC versus d4T (VL < 50–100 copies/ml)	688 per 1000	881 per 1000	RR 1.28	326	⊕ ⊕ ⊕⊝
Follow-up: mean 48 weeks		(461–1000)	(0.67–2.42	(2 studies)	moderate^a^
Adverse events	0 per 1000	0 per 1000	RR 2.41	81	⊕ ⊕ ⊝⊝
Follow-up: mean 48 weeks		(0–0)	(0.1–57.52)	(1 study)	low^a,b^
Death	0 per 1000	0 per 1000	RR 2.41	81	⊕ ⊕ ⊝⊝
Follow-up: mean 48 weeks		(0–0)	(0.1–57.52)	(1 study)	low^a,b^

^a^ Estimate of effect has a wide conference interval, including both a reduction and increase of effects

^b^ One study with a small number of participants

* The corresponding risk (and its 95% confidence interval) is based on the assumed risk in the comparison group and the relative effect of the intervention (and its 95% CI)

PENTA 2002 followed up study participants for 5 years. Those on abacavir- containing regimen likely to achieve undetectable virologic suppression than those on the zidovudine-containing regimen (RR 1.96; 95 % CI 1.11–3.34; *n* = 69).

##### CD4 cell count

Only PENTA 2002 measured CD4 cell count (absolute, cells/mL) from baseline to 48 weeks. The study authors reported that for the abacavir group, there was a median change of 272 (95 % CI 111–434; *n* = 47) while for the other NRTI group the CD4 cell count was 182 (95 % CI 2–361; *n* = 36). We did not calculate an effect size for this outcome because it was reported as median change, therefore the quality of the evidence for this outcome was not assessed.

##### Adverse events requiring treatment interruption or switching regimens

Only PENTA 2002 reported findings on adverse events that led to treatment interruption or switching of regimens. We detected no difference between the intervention and control groups (RR 2.41; 95 % CI 0.10–57.52; *n* = 81). The quality of the evidence for this outcome was considered low.

##### Hypersensitivity reaction

Musiime 2004 reported the findings of both treatment naïve and experienced cases together without disaggregation. Abacavir had no case of hypersensitivity reaction but in the control groups there were reactions (abacavir = 0/164 = 0 %, zidovudine = 1/158 = 0.63 %, stavudine = 2/156 = 1.28 %; *n* = 478). We did not calculate an effect size for this outcome because we could not obtain data for only the treatment naïve participants. Therefore the quality of the evidence for this outcome was not assessed. PENTA 2002 reported a case of abacavir hypersensitivity reaction in child on abacavir/zidovudine/nelfinavir combination. Both trials did not give any specific definition of abacavir hypersensitivity reaction and HLA-B*5701 screening test was not carried out on any of the participants.

##### Death

Musiime 2004 reported death as an outcome for both treatment naïve and experienced participants without disaggregating the two groups (*n* = 478). The authors reported 19 deaths with no significant difference across the three arms of the study without specifying the numbers for each arms of intervention. PENTA 2002 showed no difference between abacavir and zidovudine groups (RR 2.41; 95 % CI 0.10–57.52; *n* = 81). The quality of the evidence for this outcome was low as shown in Table 5.

##### Myocardial infarction and other cardiovascular events

None of the studies measured myocardial infarction and other cardiovascular outcomes.

#### Comparison 2: Abacavir-containing cART regimens compared to cART regimens containing zidovudine or stavudine (cohort studies)

##### Virologic suppression

For the included cohort studies, only Technau 2014 reported findings on virologic suppression. The study reported < 50 copies/mL as the lowest value concentration of HIV-1 RNA non-detectable level. We found abacavir regimen to be less likely to achieve virologic suppression than stavudine regimen (RR 0.79; 95 % CI 0.67–0.92; *n* = 2165) (Fig. 3). The quality of the evidence for this outcome was low as shown in Table 6.

**Table 6:** Summary of findings table for abacavir-containing cART regimens compared to cART regimens containing zidovudine or stavudine (cohort studies)

Outcomes	Illustrative comparative risks* (95 % CI)		Relative effect (95 % CI)	No of Participants (studies)	Quality of the evidence (GRADE)
	Assumed risk	Corresponding risk			
	AZT or d4T - containing combination antiretroviral regimens	ABC -containing combination antiretroviral regimens			
Virologic suppression - Lopinavir/ ritonavir based (VL < 50 copies/ml)	516 per 1000	408 per 1000	RR 0.79	2165	⊕ ⊕ ⊝⊝
Follow-up: mean 48 weeks		(346–475)	(0.67–0.92)	(1 study)	low
Virologic failure	211 per 1000	213 per 1000	RR 1.01	557	⊕ ⊕ ⊝⊝
Follow-up: mean 24 months		(154–294)	(0.73–1.39)	(1 study)	low
Death	35 per 1000	25 per 1000	RR 0.72	557	⊕ ⊕ ⊝⊝
Follow-up: mean 24 months		(9–67)	(0.27–1.92)	(1 study)	low

* The corresponding risk (and its 95% confidence interval) is based on the assumed risk in thecomparison group and the relative effect of the intervention (and its 95% CI)

##### Virologic failure

Brennan 2014 defined virologic failure as the proportion of participants with a viral load of more than 400 copies/mL after 24 months of treatment. We detected no difference in virologic failure between abacavir regimen and stavudine regimen (RR 1.01; 95 % CI 0.73–1.39; *n* = 557). The quality of the evidence for this outcome was considered low.

##### Death

For Brennan 2014 we detected no difference between abacavir and stavudine regimen at 24 months follow-up period (RR 0.72; 95 % CI 0.27–1.92; *n* = 557). The quality of the evidence for this outcome was judged low.

## Discussion

The participants of the included studies received twelve different drug combinations. Comparing effects of the interventions across the individual studies was limited due to different study designs, important outcomes not measured in all studies, statistical heterogeneity between studies, and the multiplicity of the interventions. Different drug combinations such as NRTI backbones like abacavir/lamivudine, zidovudine/lamivudine and stavudine/lamivudine; and in using efavirenz, lopinavir/ritonavir, nevirapine, and nelfinavir as the third drug; plus the use of double or triple ART combinations accounted for the multiplicity nature of the interventions.

The included studies compared abacavir/lamivudine to stavudine/lamivudine or zidovudine/lamivudine as double or triple drug regimens and used NNRTIs or PIs as a third drug. This potentially masked the head-to-head comparison of abacavir to stavudine or zidovudine. The PENTA 2002 study compared abacavir/lamivudine, zidovudine/lamivudine and abacavir/zidovudine with a PI (nelfinavir) as the third drug [31, 32]. However, only some symptom free participants were given the PI while others were on two drug regimens. Nelfinavir is found to be less efficacious than current PI regimens such as ritonavir-boosted PIs, both in tolerability and virologic suppression, and it is no longer a component of recommended ART regimen for children or adults [39]. The use of abacavir/zidovudine double therapy combination is no longer encouraged in the management of HIV infected children [4].

Findings across studies contrasted each other for the outcome virologic suppression as Musiime [28] showed no difference between abacavir-containing regimen and that of the stavudine-containing regimen. The same study found abacavir regimen to be less efficacious when compared with those containing zidovudine for up to 48 weeks of treatment. However, PENTA [32] findings showed that abacavir- containing regimen is marginally more efficacious when compared to others at 48 weeks. The cohort study by Technau and colleagues found that abacavir-containing regimens had lesser virologic efficacy in comparison to stavudine regimens for both lopinavir/ ritonavir and efavirenz based combinations [15]. Brennan 2014, on the other hand, found no difference in virologic failure between abacavir and stavudine regimens [29]. There was also no difference in incidence of death in the intervention and control groups in the PENTA 2002 and Brennan 2014 studies. Musiime and colleagues (2014) did not observe any abacavir-related hypersensitivity reactions in their trial. They found abacavir-containing regimen was well tolerated and had little safety concerns in terms of adverse events that warrants discontinuation [28]. PENTA 2002 reported a case of reaction to abacavir and three other cases that were suspected to be abacavir hypersensitivity reactions [14]. None of the included studies reported on myocardial infarction and other cardiovascular events.

Of all the included studies, the most direct comparisons between abacavir and other NRTIs in children was by the Musiime 2014 trial which used currently recommended regimens. The findings showed that treatment naïve children did well on abacavir, zidovudine and stavudine based triple regimens, with low toxicity for the three regimens and high viral load suppression up at 96 weeks follow up period. Musiime 2014 findings also support the ARROW (AntiRetroviral Research fOr Watoto) study report of low incidence of abacavir hypersensitivity reaction in African children. ARROW was an open-label randomised evaluation of induction-maintenance and monitoring strategies in symptomatic HIV-infected infants and children initiating abacavir and lamivudine plus nevirapine or efavirenz in Uganda and Zimbabwe. The low incidence of abacavir hypersensitivity reaction among the children was attributed to lower prevalence of HLA-B*5701 in African populations [12]. Brennan 2014 concluded that although abacavir and stavudine regimens had comparable viral load status by 24-months on treatment, participants on stavudine had higher risk of death and poorer immune response outcomes [29].

A systematic review and meta-regression analysis by Hill and Sawyer suggests lower efficacy for first-line use of abacavir/lamivudine NRTI backbone with boosted PIs relative to tenofovir/ emtricitabine in adults. There were also assumptions that the effect might have been confounded by differences between the trials in terms of their baseline characteristics, patient management and adherence [40]. Another systematic review and meta-analysis, by Cruciani et al., assessed treatment naïve and treatment experienced HIV-infected adult patients and found that abacavir-containing regimens and tenofovir-containing regimens have similar virological efficacy. Adverse events requiring discontinuation of treatment were reported to occur slightly more frequently in abacavir recipients but the difference was not statistically significant [41]. However, none of the intervention arms of the included studies of this systematic review has tenofovir or emtricitabine NRTI backbone to make a good comparison.

### Overall completeness and applicability of evidence

The included studies in this review have a variety of limitations. PENTA 2002 was a small (*n* = 81), open-label trial while for Musiime 2014 we only have a conference presentation to provide information about the study. Brennan 2014 was also a conference presentation with numerous issues that needed clarification, while Technau 2014 presented findings only on virologic suppression. Technau 2014 had a considerable proportion of the participants (44 %) not doing viral load testing during the 12 month window period (probably due to the fact that abacavir was recently adopted for use thereby having fewer data available unlike stavudine). There is also the possibility of selection bias if viral load testing was done in children who appear more healthy [14, 15]. Apart from PENTA 2002 that had a 5 year follow up, the other studies had rather shorter follow up periods which limits the confidence we can have in the findings.

### Quality of the evidence

The reporting of the included studies was largely inadequate and this necessitated contacting the trial authors to try and obtain the required information. Unfortunately we were unsuccessful in this regard for the studies Musiime 2014, Brennan 2014, Flynn 2014 and Technau 2014. The lead author of Technau 2014 advised that we should continue using the available data while an author from Flynn 2014 referred us to the sponsors. The sponsors, however, were reluctant to share the requested data. The virologic suppression outcome data of treatment naïve and experienced participants in the Musiime 2014 study was not disaggregated but we estimated the total number of participants for each arm by using the reported randomisation ratio of 1:1:1 to calculate the total number for each arm for the treatment naïve participants. We downgraded the evidence from the RCTs for imprecision because the estimate of effect has a wide conference interval, including both a reduction and increase of effects; and did not upgrade any of the evidence from the cohort studies. Significant heterogeneity (I^2^ = 81 % and 88 %) is probably due to the multiplicity nature of the antiretroviral combinations.

The NOS quality rating for the two observational studies was good for the Selection domains, fair for the Comparability domain and fair for the Outcome domain [42]. The biggest limitation in the cohort studies was the low uptake viral load testing between 65 and 75 % at 6 and 12 months with larger proportion of children on stavudine doing the viral load testing than those on abacavir [15].

### Potential biases in the review process

We minimised the biases in the review process by having a comprehensive search strategy across a number of databases and other sources, as well as not limiting the search to studies in specific languages. We were unable to assess the likelihood of publication bias due to having only four included studies.

## Conclusions

### Implications for practice

Abacavir-containing cART regimens remain a viable option as the first-line treatment for HIV infected children and adolescents as recommended by the WHO [4]. In this review we found that abacavir in combination with other classes of antiretroviral medicine is not different in efficacy and safety when compared to stavudine and zidovudine, for both the early stage and long term treatment. For this reason, the current clinical guideline from the WHO that recommends abacavir, stavudine or zidovudine for treating children and adolescents based on the patients’ profile in terms of toxicity and non-availability of other preferred regimens should still stand.

### Implications for policy making

The quality of the evidence available from the included studies based on GRADE criteria was generally low for the analysed outcomes except in the case of virologic suppression for the RCTs which was moderate. The findings of this study are not suggestive of any major change in the existing treatment policy.

### Implications for research

There is a need for adequately powered and well planned RCTs of abacavir-containing cART regimens that are reported according to the CONsolidated Standards of Reporting Trials (CONSORT) guidelines [43]. These studies should be designed to generate high-quality evidence in different settings. The focus should be on virologic response, adverse events (including hypersensitivity reactions and cardiovascular events) and mortality. Harm may also be researched with analytical cohort studies if more feasible.

Further research on abacavir-containing cART regimen should also be geared towards defining the subgroup of HIV infected children and adolescents for whom this regimen will be most beneficial, such as different age groups, co-morbidities and different dosages and combinations of regimens. Common comparator regimens such as those containing zidovudine, tenofovir and emtricitabine NRTI backbones should be included in the RCTs. The research studies should ideally have a follow-up duration of at least 5 years.

## Additional files

Additional file 1:
**Search strategies.** (PDF 347 kb) Additional file 2:
**Assessment of risk of bias in included RCTs.** (PDF 299 kb) Additional file 3:
**Newcastle – Ottawa Quality Assessment Scale for included Cohort Studies.** (PDF 240 kb)

## Abbreviations

ABC: Abacavir
AIDS: Acquired immunodeficiency syndrome
AZT: Zidovudine
cART: Combination antiretroviral therapy
CENTRAL: Cochrane Central Register of Controlled Trials
CROI: Conference on Retroviruses and Opportunistic Infections
DNA: Deoxyribonucleic acid
EACS: European AIDS Clinical Society
EFV: Efavirenz
FTC: Emtricitabine
GRADE: Grading of Recommendations Assessment, Development and Evaluation
HIV: Human immunodeficiency virus
IAS: International AIDS Society
MeSH: Medical subject heading
NNRTIs: Non-nucleoside reversible transcriptase inhibitors
NRTIs: Nucleoside reverse transcriptase inhibitors
NVP: Nevirapine
RevMan: Review Manager
RCT: Randomised controlled trial
d4T: Stavudine
TDF: Tenofovir
WHO: World Health Organization
