# Supplementary material for: Efficacy and safety of abacavir-containing combination antiretroviral therapy as first-line treatment of HIV infected children and adolescents: a systematic review and meta-analysis
Source: BMC Infect Dis. 2015 Oct 26;15:469. doi: 10.1186/s12879-015-1183-6 (PMC4623925; doi:10.1186/s12879-015-1183-6)
Supplement: Additional file 1: — Search strategies. (PDF 347 kb) [file 12879_2015_1183_MOESM1_ESM.pdf]

Additional file 1. Search strategies

1. Search strategy for CENTRAL

#1 (HIV INFECTIONS) OR HIV OR HIV OR HIV-1* OR HIV-2* OR HIV1 OR HIV2 OR (HIV INFECT*) OR (HUMAN IMMUNODEFICIENCY VIRUS) OR (HUMAN IMMUNEDEFICIENCY VIRUS) OR (HUMAN IMMUNO-DEFICIENCY VIRUS) OR (HUMAN IMMUNE-DEFICIENCY VIRUS) OR ((HUMAN IMMUN*) AND (DEFICIENCY VIRUS)) OR (ACQUIRED IMMUNODEFICIENCY SYNDROME) OR (ACQUIRED IMMUNEDEFICIENCY SYNDROME) OR (ACQUIREDIMMUNO-DEFICIENCY SYNDROME) OR (ACQUIREDIMMUNE-DEFICIENCY SYNDROME) OR ((ACQUIREDIMMUN*) AND (DEFICIENCY SYNDROME)) OR (VIRAL SEXUALLY TRANSMITTED DISEASES)

#2 (HIGHLY ACTIVE ANTIRETROVIRAL THERAPY) OR (ANTI-RETROVIRAL AGENTS) OR (ANTIVIRAL AGENTS) OR((ANTI) AND (HIV)) OR ANTIRETROVIRAL*OR ((ANTI) AND(RETROVIRAL*)) OR HAART OR((ANTI) AND (ACQUIREDIMMUNODEFICIENCY)) OR ((ANTI) AND (ACQUIREDIMMUNEDEFICIENCY)) OR ((ANTI) AND (ACQUIRED IMMUNO-DEFICIENCY)) OR ((ANTI) AND (ACQUIRED IMMUNE-DEFICIENCY)) OR ((ANTI) AND (ACQUIRED IMMUN*) AND (DEFICIENCY))

#3 INFAN* OR CHILD* OR ADOLESCENT*

#4 PEDIATRIC* OR PAEDIATRIC

#5 (#3 OR #4)

#6 ABACAVIR OR ZIAGEN OR 1592U89

#7 (#1 and #2 and #5 and #6)

2. Search strategy for MEDLINE via PubMed

#1 (HIV Infections[MeSH] OR HIV[MeSH] OR hiv[tw] OR hiv-1*[tw] OR hiv-2*[tw] OR hiv1[tw] OR hiv2[tw] OR hiv infect*[tw] OR human immunodeficiency virus[tw] OR human immunedeficiency virus[tw] OR human immuno-deficiency virus[tw] OR human immune-deficiency virus[tw] OR ((human immun*) AND (deficiency virus[tw])) OR acquired immunodeficiency syndrome[tw] OR acquired immunedeficiency syndrome[tw] OR acquired immuno-deficiency syndrome[tw] OR acquired immune-deficiency syndrome[tw] OR ((acquired immun*) AND (deficiency syndrome[tw])) OR "sexually transmitted diseases, viral"[MESH:NoExp])

#2 ("Antiretroviral Therapy, Highly Active"[MeSH] OR "Anti-Retroviral Agents"[MeSH] OR "Antiviral Agents"[MeSH: NoExp] OR ((anti) AND (hiv[tw])) OR antiretroviral*[tw] OR ((anti) AND (retroviral*[tw])) OR HAART[tw] OR ((anti) AND (acquired immunodeficiency[tw])) OR ((anti) AND (acquired immunedeficiency[tw])) OR ((anti) AND (acquired immuno- deficiency[tw])) OR ((anti) AND (acquired immune-deficiency[tw])) OR ((anti) AND (acquired immun*) AND (deficiency[tw])))

#3 ("child"[MeSH Terms] OR "child"[All Fields]) OR ("infant"[MeSH Terms] OR "infant"[All Fields]) OR ("adolescent"[MeSH Terms] OR "adolescent"[All Fields])

#4 ("pediatrics"[MeSH Terms] OR "pediatrics"[All Fields] OR "pediatric"[All Fields]) OR ("pediatrics"[MeSH Terms] OR "pediatrics"[All Fields] OR "paediatric"[All Fields])

#5 (#3 OR #4)

#6 (abacavir OR ziagen OR 1592U89)

#7   (#1 AND #2 AND #5 AND #6)

3. Search strategy for Scopus

#1 (“hiv infection*” OR hiv OR "hiv-1" OR "human immunodeficiency virus" OR "human immunedeficiency virus")

#2 (“acquired immune-deficiency syndrome” OR “acquired immunodeficiency syndrome” OR aids)

#3   (#1 OR #2)

#4 ("highly active antiretroviral therapy" OR haart OR "antiretroviral therapy" OR antiretrovir* OR "antiretroviral agents")

#5 (abacavir OR ziagen OR 1592u89)

#6 (child* OR infant* OR pediatric* OR paediatric* OR adolescent*)

#7 (#3 AND #4 AND #5 AND #6)

4**. Search string for ISI Web of Science**

(HIV OR HIV/AIDS OR human immun* OR acquired immun*)
(antiretroviral therapy OR anti-retroviral therapy OR ARV OR HAART)
(abacavir OR ziagen OR 1592U89)
(child* OR infant* OR pediatric* OR paediatric* OR adolescent*)

TOPIC: ((HIV OR HIV/AIDS OR human immun* OR acquired immun*)) *AND* **TOPIC:** ((antiretroviral therapy OR anti-retroviral therapy OR ARV OR HAART)) *AND* **TOPIC:** ((abacavir OR ziagen OR 1592U89)) *AND* **TOPIC:** ((child* OR infant* OR pediatric* OR paediatric* OR adolescent*))
