# Supplementary material for: Efficacy and safety of abacavir-containing combination antiretroviral therapy as first-line treatment of HIV infected children and adolescents: a systematic review and meta-analysis
Source: BMC Infect Dis. 2015 Oct 26;15:469. doi: 10.1186/s12879-015-1183-6 (PMC4623925; doi:10.1186/s12879-015-1183-6)
Supplement: Additional file 2: — Assessment of risk of bias in included RCTs. (PDF 299 kb) [file 12879_2015_1183_MOESM2_ESM.pdf]

Additional file 2. Assessment of risk of bias in included RCTs

Domain 1: sequence generation

Adequate: investigators described a random component in the sequence generation process such as the use of:

• a random number table;

• coin tossing;

• throwing dice;

• shuffling cards or envelopes.

Inadequate: investigators described a non-random component in the sequence generation process such as the use of:

• odd or even date of birth;

• the day or date of admission;

• the hospital or clinic record number;

• preference of the participant;

• the results of a laboratory test or series of tests.

Unclear: there is insufficient information to permit judgement of the way in which sequence generation was performed.

Domain 2: allocation concealment

Adequate: neither participants nor investigators enrolling participants could foresee assignment due to:

• central allocation (e.g. via the telephone or pharmacy-controlled);

• sequentially numbered drug containers of a matching appearance;

• sequentially numbered, opaque and sealed envelopes.

Inadequate: both participants and investigators enrolling participants could foresee upcoming assignment based on, for example:

• using an open random allocation schedule;

• assigned envelopes were unsealed, non-opaque or not numbered appropriately;

• date of birth;

• case record number.

Unclear: there is insufficient information to permit judgement to the sequence generation process.

Domain 3: blinding

Adequate: when any one of the following are applicable:

• no blinding, but the review authors judge that the outcome would not be influenced by a lack of blinding;

• blinding of both the key study personnel and participants are ensured, and it is unlikely that blinding could have been broken;

• either participants or some key study personnel were not blinded, but the outcome measurement was blinded and the non-blinding of others are not likely to introduce bias.

Inadequate: when any one of the following is applicable:

• no blinding or incomplete blinding;

• blinding of key study personnel and participants were attempted, but it is likely that the blinding could have been broken;
• either key study personnel or participants were not blinded, which is likely to introduce bias.
Unclear: there is insufficient information to permit judgement, or the study did not address this outcome at all.
Domain 4: incomplete outcome data
Adequate: when any one of the following is applicable:
• no missing outcome data;
• the reasons for missing outcome data are unlikely to be related to the true outcome;
• missing outcome data are balanced in numbers across intervention groups;
• missing data have been imputed using appropriate methods;
• for dichotomous data, the proportion of missing outcomes compared with the observed event risk is not enough to have a clinically relevant impact on the intervention effect estimate;
• for continuous data, the plausible effect size among missing outcomes is not enough to have a clinically relevant impact on the observed effect size.
Inadequate: when any one of the following is applicable:
• the reasons for missing outcome data are likely to be related to true outcome;
• the application of simple imputation is potentially inappropriate;
• 'as-treated' analysis done with substantial departure of the intervention received from that assigned at randomisation;
• for dichotomous data, the proportion of missing outcomes compared with the observed event risk is enough to introduce clinically relevant bias in the intervention effect estimate;
• for dichotomous outcome data, the plausible effect size among missing outcomes is enough to induce clinically relevant bias in the observed effect size.
Unclear: there is insufficient reporting of exclusions to permit judgement, or the study did not address this outcome at all.
Domain 5: selective outcome reporting
Adequate: when any one of the following is applicable:
• the study protocol is available and all of the prespecified outcomes are addressed in the review in the prespecified way;
• the study protocol is not available, but it is clear that the published reports include all the prespecified and expected outcomes.
Inadequate: when any one of the following is applicable:
• not all of the prespecified primary outcomes have been reported;
• one or more of the primary outcomes is reported using measurements of analysis methods that were not prespecified;
• one or more reported primary outcomes were not prespecified;
• one or more outcomes of interest in the review are reported incompletely so that they cannot be entered in a meta-

analysis;

• the study report fails to include results for a key outcome that would be expected to have been reported for such a study.

Unclear: there is insufficient information to permit judgement of compliance.

Domain 6: other potential threats to validity

Adequate: when the study seems to be free of other sources of bias.

Inadequate: when there is the possibility of at least one important risk of bias such as:

• the quality of the specific study design is in question;

• the study is stopped early due to some data-dependent process;

• the study has been claimed to have been fraudulent.

Unclear: when there may be a risk of bias, but there is either:

• insufficient information to assess whether an important risk of bias exists;

• insufficient rationale or evidence that an identified problem will introduce bias.
