# Supplementary material for: Efficacy and safety of abacavir-containing combination antiretroviral therapy as first-line treatment of HIV infected children and adolescents: a systematic review and meta-analysis
Source: BMC Infect Dis. 2015 Oct 26;15:469. doi: 10.1186/s12879-015-1183-6 (PMC4623925; doi:10.1186/s12879-015-1183-6)
Supplement: Additional file 3: — Newcastle – Ottawa Quality Assessment Scale for included Cohort Studies. (PDF 240 kb) [file 12879_2015_1183_MOESM3_ESM.pdf]

Additional file 3. Newcastle – Ottawa Quality Assessment Scale Cohort Studies

Note: A study can be awarded a maximum of one star for each numbered item within the Selection and Outcome categories. A maximum of two stars can be given for Comparability.

Selection

1) Representativeness of the exposed cohort
   a) truly representative of the average _______________ (describe) in the community ❋
   b) somewhat representative of the average ______________ in the community ❋
   c) selected group of users eg nurses, volunteers
   d) no description of the derivation of the cohort

2) Selection of the non-exposed cohort
   a) drawn from the same community as the exposed cohort❋
   b) drawn from a different source
   c) no description of the derivation of the non-exposed cohort

3) Ascertainment of exposure
   a) secure record (eg surgical records) ❋
   b) structured interview❋
   c) written self-report
   d) no description

4) Demonstration that outcome of interest was not present at start of study
   a) yes ❋
   b) no

Comparability

1) Comparability of cohorts on the basis of the design or analysis
   a) study controls for _____________ (select the most important factor) ❋
   b) study controls for any additional factor ❋ (This criteria could be modified to indicate specific control for a second important factor.)

Outcome

1) Assessment of outcome
   a) independent blind assessment ❋
   b) record linkage ❋
   c) self-report
   d) no description

2) Was follow-up long enough for outcomes to occur
   a) yes (select an adequate follow up period for outcome of interest) ❋

b) no

3) <u>Adequacy of follow up of cohorts</u>
a) complete follow up - all subjects accounted for ✵
b) subjects lost to follow up unlikely to introduce bias - small number lost - > ____ % (select an adequate %) follow up, or description provided of those lost) ✵
c) follow up rate < ____% (select an adequate %) and no description of those lost
d) no statement
